# Associations of cardiac autonomic modulation and urinary adrenal hormones with marathon finishing time in middle-aged male athletes

**DOI:** 10.3389/fcvm.2026.1932814

**Published:** 2026-10-12

**Authors:** Amedeo De Antoni, Lucia Silvia Giobbi, Sergei Bondarev, Martina Calligione, Francesca Graziano, Simone Ungaro, Matteo Pizzolato, Alessandro Gianni, Giulia Pivetta, Lorenzo Da Rin De Monego, Girolamo Architetto, Giorgia Antonelli, Giovanna Tretti, Filippo Ceccato, Angelica Carandina, Ludovico Furlan, Greta Salafia, Francesca Magna, Thais Marques da Silva, Martina Montagnana, Nicola Montano, Alessandro Zorzi, Eleonora Tobaldini

**Affiliations:** 1 Department of Cardiac, Thoracic and Vascular Sciences and Public Health, University of Padua, Padua, Italy; 2 Department of Internal Medicine, Fondazione IRCCS Ca’ Granda Ospedale Maggiore Policlinico, Milan, Italy; 3 Department of Sports Medicine, Semmelweis University, Budapest, Hungary; 4 Department of Medicine DIMED, University of Padova, Padua, Italy; 5 Laboratory Medicine Unit, University Hospital of Padova, Padua, Italy; 6 Unit of Endocrinology, University-Hospital of Padova, Padua, Italy; 7 Department of Physiology, Ribeirão Preto Medical School, University of São Paulo, São Paulo, Brazil; 8 Department of Clinical Sciences and Community Health, Dipartimento di Eccellenza 2023–2027, University of Milan, Milan, Italy; 9 Ospedale Riabilitativo di Alta Specializzazione (ORAS), Motta di Livenza, Italy

**Keywords:** adrenal hormones, athletic performance, autonomic nervous system, heart rate variability (HRV), overtraining

## Abstract

**Background:**

The autonomic nervous and endocrine systems are involved in the physiological adaptation to exercise, and in experimental settings both the sympathovagal balance and the adrenal function are associated with athletic performance. In this real-life field study, the association between non-invasive markers of cardiac autonomic modulation, urinary adrenal hormone levels, and marathon finishing time in middle-aged Caucasian male athletes was investigated.

**Methods:**

A prospective study was conducted in male marathon runners aged ≥30 years. Continuous single-lead electrocardiographic (ECG) recordings were obtained using a wearable chest device from the day before the marathon until after its completion. Urinary adrenal hormone levels were measured before and after the race. Univariate linear regression analyses were performed to correlate general characteristics, nocturnal heart rate variability (HRV) parameters, and hormonal parameters with marathon finishing time. A multivariable model was then applied including age, body mass index (BMI), and training hours.

**Results:**

104 athletes were enrolled in the study and 73 (median age 48 years, interquartile range = 16) had adequate data quality for the final analysis. Several HRV parameters correlated with better performance at univariate analysis; lower minimum nocturnal heart rate and vagal predominance, expressed as higher 2 Like Variations (2LV) values remained independently associated with shorter finishing time. Among hormonal variables, the post-/pre-race ratio of urinary normetanephrine hourly output and cortisol/cortisone ratio remained significant at multivariable analysis. Only BMI, but not age and training history, remained significant in a model integrating HRV and hormones.

**Conclusions:**

Under real-world marathon conditions, specific HRV indices and endocrine markers showed promising value as indicators of physiological readiness with endurance exercise in male master athletes.

## Graphical Abstract

**Figure:**
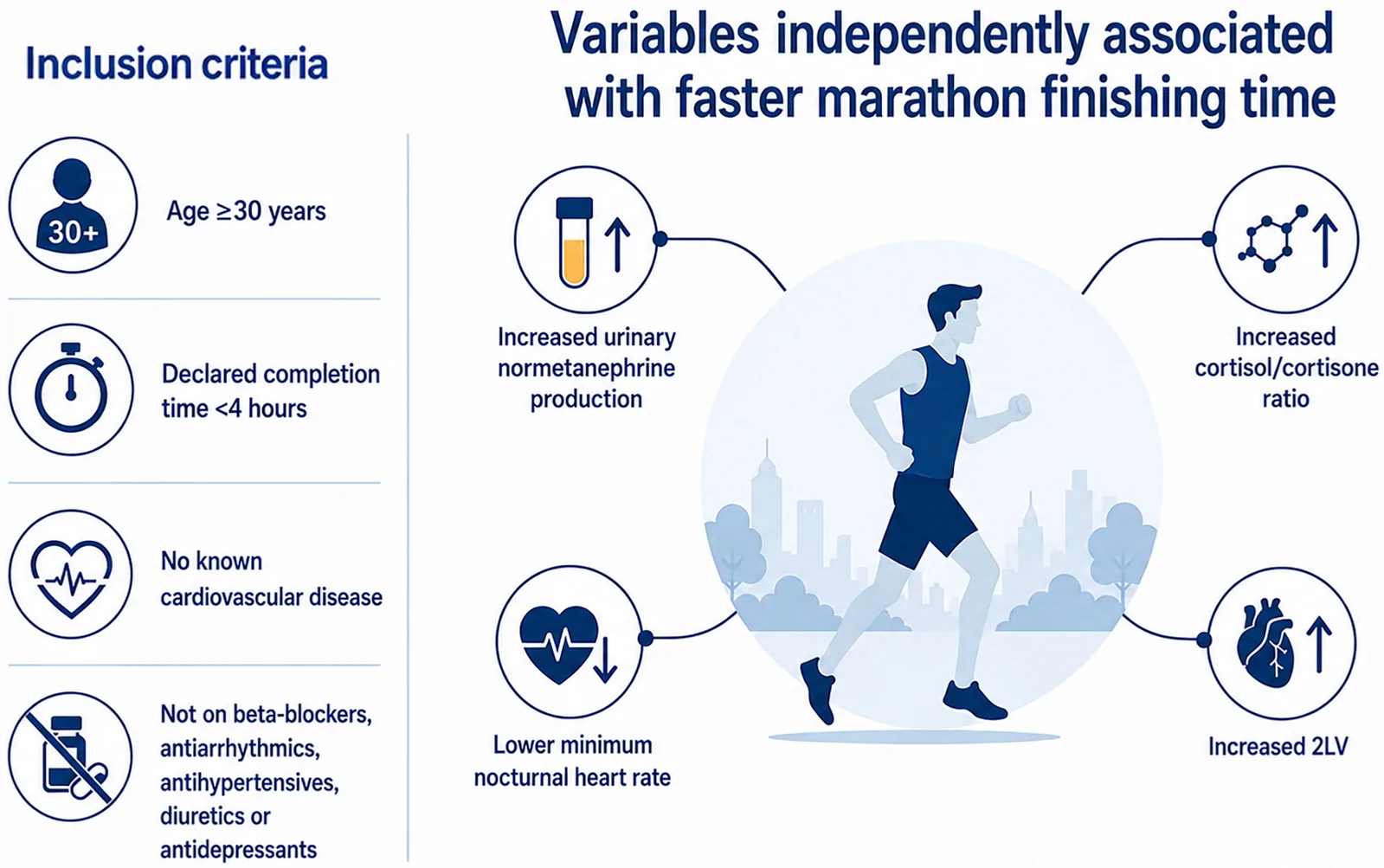

## 1 Introduction

In recent years, the growing interest in physical activity and the increasing participation in endurance sports have stimulated the search for reliable indicators of wellness, training status, and athletic performance. In this context, the autonomic nervous system (ANS) has gained particular relevance, as the dynamic balance between its sympathetic and parasympathetic components plays a key role in cardiovascular regulation and exercise adaptation (1–4).

Autonomic function is commonly assessed through heart rate variability (HRV), a non-invasive marker of cardiac autonomic modulation that reflects the dynamic interaction between sympathetic and parasympathetic influences on the sinus node (1–3). At rest, enhanced parasympathetic modulation, as assessed by vagal-related HRV indices, has been consistently associated with favourable training adaptations, including lower resting heart rate, increased stroke volume, and improved cardiac metabolic efficiency (2, 5). For this reason, resting HRV has been widely proposed as a marker of training status and readiness for performance (1–4). Beyond autonomic regulation, endurance exercise elicits a complex endocrine response, primarily involving activation of the hypothalamic–pituitary–adrenal (HPA) axis and the sympathetic–adrenal system (3–6). Both glucocorticoid metabolism and catecholamine excretion have also been associated with endurance performance in trained athletes (7, 8).

Taken together, HRV and endocrine markers appear to be promising tools for monitoring training load, recovery, and the risk of overtraining, a condition that may progress to maladaptive physiological responses if not adequately recognised (1, 2, 7, 9, 10). Nevertheless, their accuracy, robustness, and practical applicability in endurance athletes remain incompletely defined. Importantly, most available evidence derives from controlled experimental protocols and relatively small cohorts while data obtained under real-life conditions, such as competitive endurance events, are still limited. In particular, the role of HRV as a practical marker of performance in real-world endurance exercise, and the extent to which adrenal hormones influence basal autonomic regulation and contribute to endurance performance, remain largely unexplored.

Therefore, the aim of the present study was to investigate the relationship between HRV and athletic performance, as well as the contribution of adrenal hormones, in male master athletes running a marathon.

## 2 Methods

### 2.1 Study design and participants

This was a prospective, observational field study conducted in the setting of the Padua Marathon 2025, held on April 27th, 2025. In the weeks preceding the event, Italian male master athletes aged ≥30 years and with a self-declared expected finishing time <4 h were invited to participate in the study via an e-mail sent by the Race Organization. The reason for choosing male master athletes is the need to study a physiologically homogeneous cohort. In addition, in female athletes, hormonal fluctuations related to the menstrual cycle may further affect endocrine responses and autonomic regulation, representing an additional potential source of biological variability. Although no ethnic background was pre-specified, all athletes who volunteered to participate were Caucasian.

Eligible participants were required to be free from known cardiovascular diseases and not to be taking any medications known to affect autonomic nervous system function or adrenal hormone secretion, including beta-blockers, antiarrhythmic agents, antidepressants, or anxiolytics. Of note, Italian athletes running a marathon are required by Law to undergo a pre-participation medical check including history, physical examination, resting electrocardiogram (ECG), exercise testing, spirometry and urine analysis.

Participants were instructed to record the time of their last bladder voiding before sample collection. The day preceding the race, participants were asked to void their bladder in the provided container which was weighed, and aliquots were obtained for adrenal hormone analyses. At the same time, a continuous electrocardiographic monitoring system was applied. Anthropometric data (height and body weight) and training-related information, including total years of training, personal best marathon time, and average weekly training volume (expressed as mean weekly hours of training in the last year) were collected.

On race day, participants were instructed to empty their bladder immediately before the start and to record the time. A clean container was provided to collect any urine passed during the race if needed. Immediately after finishing the marathon, participants were asked to void into the container and report to the study stand located in the finish area, where urine was weighed and a second set of samples for post-race hormonal analyses was obtained. Continuous ECG monitoring was then removed. Information regarding the intake of caffeine or other xanthines during the race was collected.

The study protocol was approved by the local Ethics Committee of the Province of Padua (6130/U6/249), and all participants provided written informed consent in accordance with the Declaration of Helsinki.

### 2.2 Electrocardiographic monitoring

Continuous electrocardiographic (ECG) monitoring was performed using a single-lead wearable patch device (ROOTi-Rx, Rooti Labs, Taiwan), which allows continuous prolonged ECG recording under real-life conditions without conventional cable-based Holter systems. The system is waterproof and has an ECG sample rate of 500 Hz.

The ECG patch was applied at the time of bib collection on the day preceding the marathon and remained in place until completion of the race, providing continuous ECG recording throughout the pre-race period and the competition.

ECG signals were initially processed using the device's built-in artificial-intelligence–based algorithm for noise reduction and artefact suppression. Subsequently, all ECG recordings were manually reviewed by trained investigators to verify signal quality and ensure the availability of high-quality segments suitable for heart rate variability analysis, which required optimal signal fidelity, particularly during nocturnal recordings.

### 2.3 Heart rate variability analysis

The ECG and actigraphy biosignals acquired from monitoring via patch device were processed by artificial intelligence (AI; Rooti Labs, Taipei City, Taiwan) and revised by an expert to obtain indexes of cardiac autonomic control during the subjects’ entire night of sleep. It was possible to obtain the tachogram (i.e., the time trend of the R–R interval series) from ECG signals by identifying and detecting of QRS complex peaks using parabolic interpolation. The R–R interval represents the elapsed time between the peaks of consecutive R waves within the QRS complex. AI was also used in ECG monitoring primarily for the detection and correction of issues within the R–R time series, which are essential for accurate heart rate analysis. Common correction methods in this context include deletion, linear and non-linear interpolation, moving average windows and non-linear predictive interpolation (11). These validated methods are designed to address problems such as ectopic beats, noise, and non-uniform sampling of R–R intervals, which can affect the accuracy of the ECG signals.

Artifact-free R–R time series recorded during the entire night of sleep were used for different approaches to HRV analysis: linear analysis in time and frequency domains, nonlinear symbolic analysis and heart rate fragmentation analysis. It is important to specify that the recording covered the entire night (approximately 6–7 h or more), therefore the analysis was performed by segmenting the signal into windows of 300 beats (corresponding to approximately 5 min) with a 50% overlap between consecutive segments. Spectral, symbolic, and time-domain HRV analyses were performed using the PyBioS Biomedical Signal Analysis and validated software (Version 1.4.1, 2021) (12).

#### 2.3.1 Linear analysis

The time-domain indices included: the standard deviation of all R–R intervals (SDNN), the root mean square of successive differences between adjacent R–R intervals (RMSSD). These indices are considered markers of global variability and vagal modulation, respectively (13).

For spectral analysis, an autoregressive model was used to extract the rhythmic oscillations characterizing the R–R time series. The Total Power was calculated, representing the overall autonomic modulation of the cardiovascular system. Additionally, three main components of the total spectrum were identified:

Very Low Frequency (VLF): frequency band <0.04 Hz;
Low Frequency (LF): frequency band between 0.04 and 0.15 Hz, reflecting both sympathetic and vagal modulation;
High Frequency (HF): frequency band between 0.15 and 0.40 Hz, reflecting parasympathetic modulation and synchronized with respiratory activity.

The power of the LF and HF components was expressed in normalized units (LF n.u. and HF n.u.), obtained by dividing the power of each band by the total power minus the VLF component (<0.04 Hz) (14, 15).

#### 2.3.2 Nonlinear analysis

Nonlinear analysis was performed using symbolic dynamics on the same segments identified for spectral analysis. To perform symbolic analysis, the R–R time series was converted into a sequence of symbols, which was divided into 3-beat patterns. These patterns were classified into four families: Zero variation (0 V): patterns with no variation; all three symbols are equal (e.g., 4-4-4);

One Variation (1 V): patterns with one variation; two consecutive symbols are equal forming a 2-beat plateau, while the remaining one is different (e.g., 2-2-5);
Two Like Variation (2LV): patterns with two like variations; all symbols are different from the previous one and arranged in ascending or descending order (e.g., 1-3-4);
Two Unlike Variation (2UV): patterns with two unlike variations; all symbols are different from the previous one but not in a consecutive order (e.g., 2-5-1).

The percentage of 0 V patterns is considered a marker of cardiac sympathetic modulation, whereas 2UV and 2LV patterns are markers of cardiac vagal modulation (16). This approach allows the assessment of non-reciprocal changes in sympathetic and parasympathetic modulation in heart period time series under physiological and pathological conditions (16, 17).

#### 2.3.3 Heart rate fragmentation

Heart rate fragmentation (HRF) is a new approach that extracts information from the dynamics of heartbeat intervals. It is proposed to evaluate ultra-rapid variability of heart rate and it has been shown to increase especially with the aging process (18–20). The analysis consisted of mapping the R–R time series to a ternary symbolic signal, attributing the symbol “−1”, “0” or “1” when the difference between successive R–R intervals was positive, null, or negative, respectively. An inflection point is represented by two different consecutive symbols, and it means a change in the direction of heart rate variation. The total percentage of inflection points (PIP) was calculated as the number of inflection points divided by the total number of valid R–R intervals in the analysed segment. Higher PIP values reflect a greater degree of fragmentation in the signal, suggesting more frequent ultra-short-term fluctuations and a more erratic heart rhythm, which may represent a pre-arrhythmic or potentially pro-arrhythmic marker (18, 19).

### 2.4 Hormone analyses

Pre- and post-race urine samples were analysed for cortisol, cortisone, aldosterone, adrenaline, noradrenaline, metanephrine, and normetanephrine. All biochemical analyses were carried out in an ISO15189-accredited clinical laboratory (21). Urinary cortisol and cortisone were determined by a liquid chromatography coupled to tandem mass spectrometry laboratory developed test, as previously described in detail (22, 23). This method was validated according to the ISO 15189 technical requirements of method validation. Urinary adrenaline and noradrenaline were measured by a CE-IVD liquid-chromatography method with electrochemical detection (ref. 6000, Chromsystems Instruments & Chemical GmbH, Germany). Urinary metanephrine and normetanephrine were quantified by a CE-IVD liquid-chromatography method with electrochemical detection (ref. 2020, Chromsystems Instruments & Chemical GmbH, Germany). Both methods were verified according to the ISO 15189 technical requirement of method verification. Urinary aldosterone was analysed with using a CE-IVD immunometric method, according to the manufacturer's instructions (ref. 310450 Liaison Aldosterone, Diasorin Italy).

Hormone concentrations were expressed either as the ratio of hormone to urinary creatinine or as an hourly production rate. The hourly production was calculated by determining the time elapsed between the previous and current void, converting the urine weight into volume using urine density, and thus calculating the total hormone amount per hour.

### 2.5 Statistical analysis

Categorical variables were expressed as numbers and percentages, while continuous variables were reported as median (interquartile range) because their distribution was not normal. The primary endpoint was the marathon completion time. The non-parametric Spearman coefficient was calculated to correlate general characteristics, nocturnal HRV parameters, and hormonal parameters with marathon time. Univariate linear regression models were used to assess the association between general, hormonal and HRV variables and finish times. Multivariable models were then constructed, including hormonal variables that were significant in the univariate analysis as well as age, body mass index (BMI), and training hours considered *a priori* potential influencers of the model. To minimise multicollinearity, multiple HRV variables were not entered simultaneously into the multivariable model. Instead, the two autonomic variables that yielded the best model fit, as indicated by the highest *R*^2^, namely minimum nocturnal heart rate and 2LV, were selected. A *p*-value <0.05 was considered significant. Statistical analyses were performed using Jamovi ver. 2.6.44.

## 3 Results

### 3.1 Breakdown and general characteristics

A total of 104 recreational male master athletes participating in the 2025 Padova Marathon, all Caucasian, agreed to take part in the study. Two athletes did not complete the race and were therefore excluded from the analysis. Among the 102 athletes who finished the marathon, 2 were unable to provide adequate post-race urine samples, and 27 had ECG recordings of insufficient quality for HRV analysis. Consequently, 73 subjects were included in the final analysis (Figure 1).

**Figure 1:**
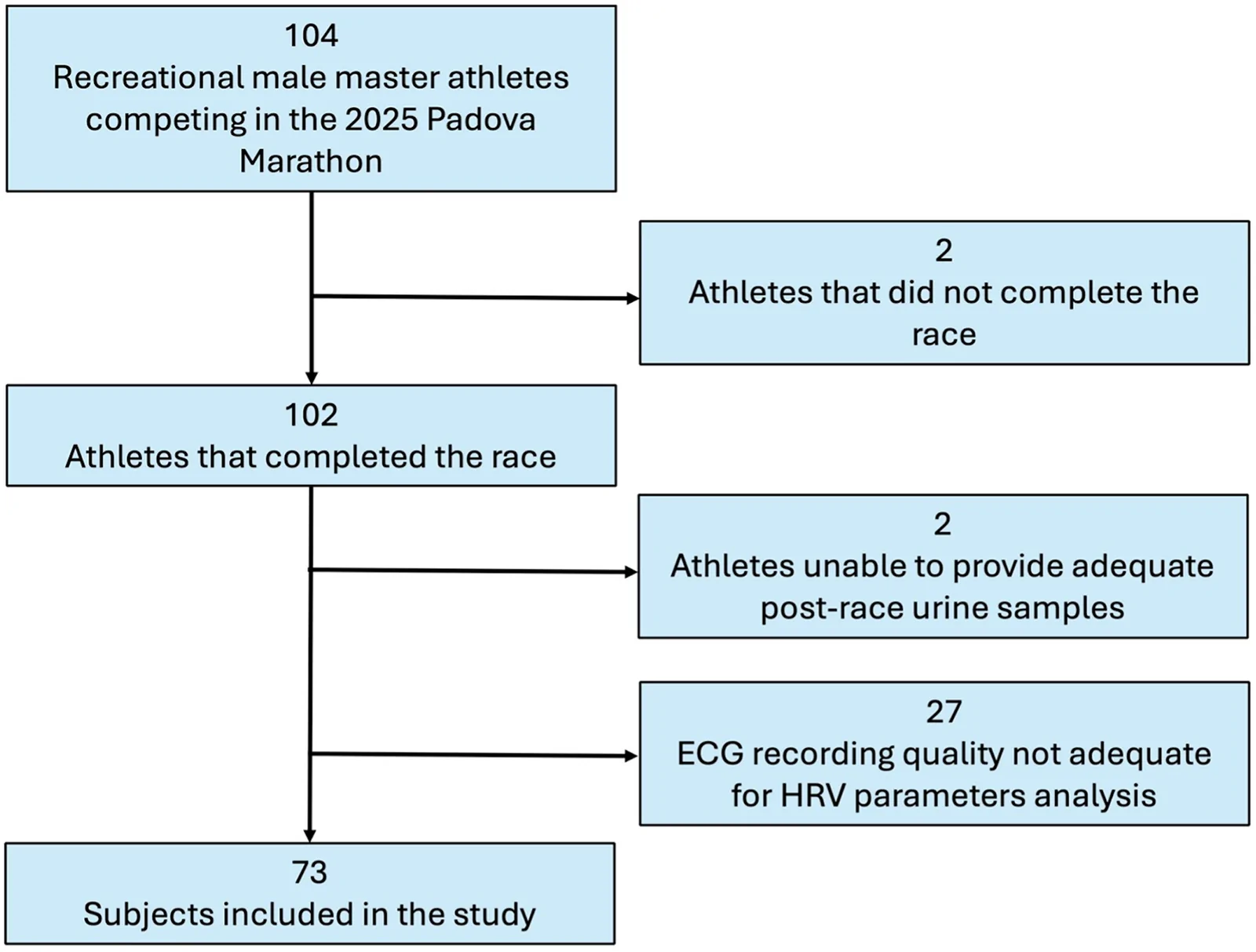
Breakdown of the study population.

Included participants had a median age of 48 (interquartile range = 16) years and a median BMI of 22.3 (1.9) kg/m^2^. They had been engaged in endurance training for a median of 10 (8) years, with a median weekly training volume of 6 (2.75) h. As per study design, all included participants were free from known cardiovascular disease and were not taking medications that could interfere with the analysis, such as antiarrhythmic drugs, antidepressants, or anxiolytics.

### 3.2 Basal characteristics and training load

Regarding baseline characteristics, younger age (*r* = 0.46 *p* < 0.001), lower BMI (*r* = 0.23, *p* = 0.03) and shorter training history (*r* = 0.26, *P* = 0.03) were associated with shorter marathon completion time while weekly training volume did not reach significance (Table 1).

**Table 1:** Spearman correlation and univariate regression analysis for association between basal characteristics and lower marathon completion time.

Variable	Median (IQR)	Correlation Rho (p)	Linear regression R (95% CI, *p*)
Age	48 (16)	0.40 (*p* < 0.001)	0.46 (0.25–0.67, *p* < 0.001)
BMI	22.3 (1.9)	0.47 (*p* < 0.001)	0.23 (0.1–0.46, *p* = 0.03)
Hours of training/week	6 (2.75)	−0.31 (*p* = 0.009)	−0.21 (−0.49 to 0.02, *p* = 0.07)
Years of sports activity	10 (8)	0.04 (*p* = 0.73)	0.26 (0.03–0.49, *P* = 0.03)

BMI, body mass index; CI, confidence interval; IQR, interquartile range.

### 3.3 Autonomic modulation indexes

At univariate analysis, lower median nocturnal heart rate and lower minimum nocturnal heart rate were associated with faster marathon completion times. In symbolic HRV analysis, lower 0 V (Spearman's *ρ* = 0.33, *p* = 0.005) and higher 2LV (Spearman's *ρ* = −0.47, *p* < 0.001) values were associated with faster marathon completion times (Figure 2). Among the other HRV parameters, RMSSD was significantly associated with faster completion times (Spearman's *ρ* = −0.36, *p* = 0.001), whereas SDNN showed a borderline association (Spearman's *ρ* = −0.22, *p* = 0.06) (Table 2).

**Figure 2:**
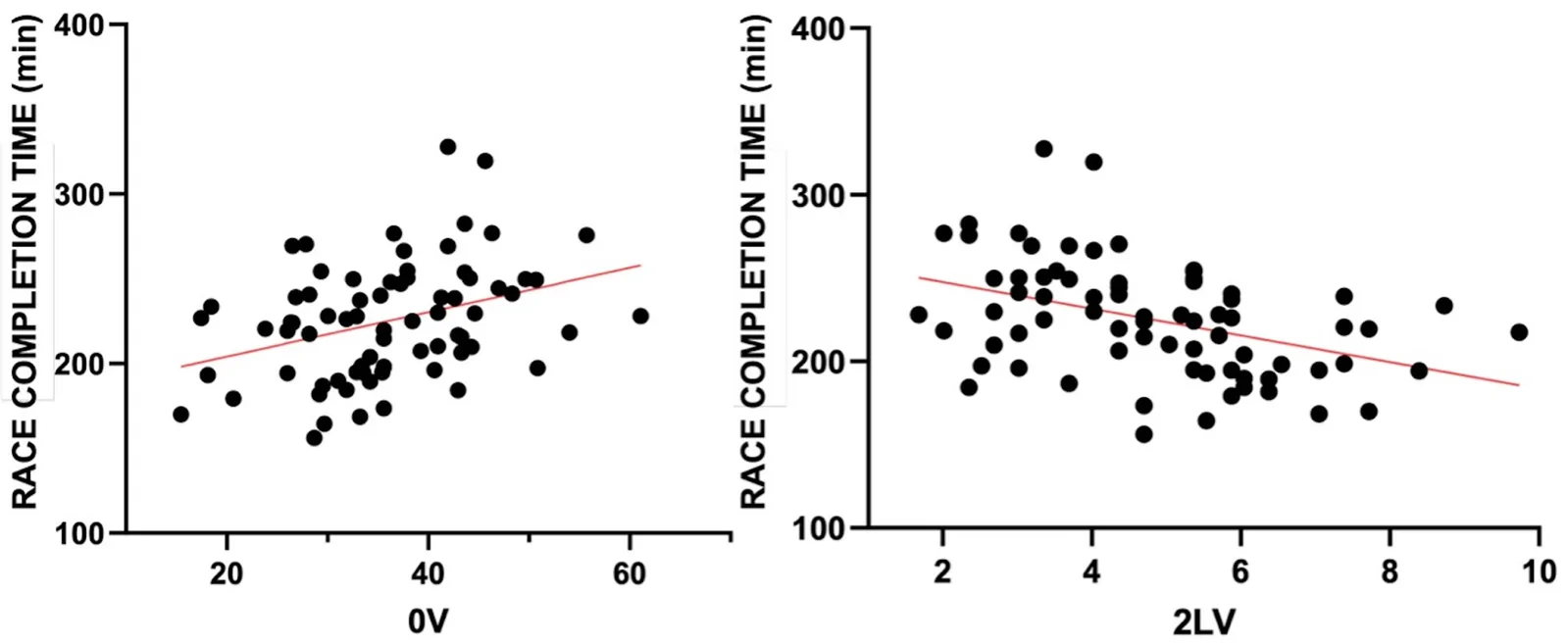
Correlation between 0 V (left) and 2LV (right) and marathon completion time. 2LV, 2 like variations; 0V, no variations.

**Table 2:** Spearman correlation and univariate regression analysis for association between HRV parameters and lower marathon completion time.

Variable	Median (IQR)	Correlation Rho (p)	Linear regression R (95% CI, *p*)
Mean Nocturnal HR (bpm)	51 (7.5)	0.38 (*p* < 0.001)	0.36 (0.13–0.59, *p* = 0.002)
Minimum Nocturnal HR (bpm)	43 (7)	0.47 (*p* < 0.001)	0.48 (0.27–0.69, *p* < 0.001)
Percentage of inflection points (PIP)	62 (9)	0.17 (*p* = 0.17)	0.17 (−0.07 to 0.40, *p* = 0.17)
LFnu	76 (15)	0.14 (*p* = 0.24)	0.18 (−0.05 to 0.42, *p* = 0.13)
HFnu	24 (15)	−0.14 (*p* = 0.24)	−0.18 (−0.42 to 0.05, *p* = 0.13)
LF/HF	3.2 (2.2)	0.14 (*p* = 0.23)	0.14 (−0.10 to 0.38, *p* = 0.14)
0V%	36 (13)	0.33 (*p* = 0.005)	0.33 (0.10–0.55, *p* = 0.005)
2LV%	4.7 (2.5)	−0.47 (*p* < 0.001)	−0.42 (−0.64 to −0.20, *p* < 0.001)
2UV%	13 (5.6)	−0.15 (*p* = 0.21)	−0.15 (−0.39 to 0.08, *p* = 0.20)
SDNNms	73 (28)	−0.22 (*p* = 0.06)	−0.24 (−0.48 to −0.01, *p* = 0.04)
RMSSDms	38 (19)	−0.36 (*p* = 0.001)	−0.35 (−0.57 to −0.12, *p* = 0.003)

IQR, interquartile range; 0V, 0 variations; 2LV, 2 like variations; 2UV, 2 unlike variations; bpm, beats per minute; HR, heart rate; HFnu, high frequency normalised units; LFnu, low frequency normalised units; LF/HF, low frequency/high frequency ratio; SDNN, standard deviation of normal-to-normal intervals; RMSSD, root mean square of successive differences.

### 3.4 Adrenal hormones

Pre- and post-race adrenal hormone concentration, expressed as the ratio of hormone to urinary creatinine or as an hourly production rate, are shown in Table 3. In particular, cortisol levels increased during the race, resulting in an increase in the cortisol/cortisone ratio. Aldosterone levels also increased in the post-race analysis. Regarding catecholamines, urinary levels of adrenaline and noradrenaline, as well as those of their metabolites, markedly increased after the race. As reported in Table 4, the post/pre race Cortisol/Cortisone ratio (*r* = −0.36, *p* = 0.002) and post/pre-race normetanephrine ratio expressed as hourly excretion (*r* = −0.27, *p* = 0.02) were significantly associated with faster Marathon completion. No other hormonal variables were significantly associated with marathon finishing time in the univariable linear regression analyses.

**Table 3:** Pre- and post-race urinary hormonal concentration (absolute values and corrected for creatinine concentration) and hourly output.

Analyte	Absolute concentration (nmol/L)		Concentration/creatinine (nmol/mmol)		Hourly output (nmol/h)	
	Median	IQR	Median	IQR	Median	IQR
Steroids						
Basal cortisol	53	57	8.79	10.7	4.08	3.91
Basal cortisone	175	92	25.6	20.8	12.5	9.2
Basal Cortisol/Cortisone ratio	0.35	0.19				
Post cortisol	220	213	16.2	19.4	13.2	12.7
Post cortisone	299	147	23.6	14.0	17.9	8.8
Post Cortisol/Cortisone ratio	0.76	0.57	—		—	
Aldosterone						
Basal aldosterone	13.2	15.5	1.83	1.48	0.996	0.775
Post aldosterone	74.8	85.1	5.18	4.11	4.49	5.11
Catecholamines						
Basal adrenaline	26.2	33.1	4.67	5.13	2.46	2.87
Basal noradrenaline	161	158	23.3	14.7	12.2	7.09
Basal metanephrine	0.120	0.115	0.019	0.018	0.009	0.009
Basal normetanephrine	0.240	0.185	0.0268	0.0232	0.015	0.012
Post adrenaline	191	203	14.6	14.5	11.5	12.2
Post noradrenaline	1,022	927	73.0	37.0	61.3	55.6
Post metanephrine	0.250	0.235	0.021	0.019	0.015	0.014
Post normetanephrine	0.760	0.480	0.059	0.034	0.046	0.029

**Table 4:** Spearman correlation and univariate regression analysis for association between hormones concentration, corrected for creatinine concentration or hour output, and finishing times.

Analyte	Concentration/creatinine		Hourly output	
	Correlation (rho, *p*)	Linear regression R (95% CI, *p*)	Correlation (rho, *p*)	Linear regression R (95% CI, *p*)
Steroids				
Basal cortisol	0.01 (*p* = 0.93)	0 (−0.25 to 0.23, *p* = 0.94)	0.06 (*p* = 0.64)	0.15 (−0.09 to 0.39, *p* = 0.23)
Basal cortisone	−0.14 (*p* = 0.23)	−0.19 (−0.43 to 0.05, *p* = 0.11)	−0.04 (*p* = 0.73)	0.02 (−0.23 to 0.26, *p* = 0.89)
Basal Cortisol/Cortisone	0.11 (*p* = 0.37)	0.22 (−0.02 to 0.45, *p* = 0.07)		
Post/Basal cortisol ratio	−0.27 (*p* = 0.02)	−0.08 (−0.32 to 0.16, *p* = 0.50)	−0.29 (*p* = 0.02)	−0.08 (−0.33 to 0.16, *p* = 0.51)
Post/Basal cortisone ratio	−0.05 (*p* = 0.71)	−0.03 (−0.27 to 0.20, *p* = 0.78)	−0.13 (*p* = 0.30)	−0.08 (−0.33 to 0.16, *p* = 0.49)
Post/Basal Cortisol/Cortisone ratio	−0.39 (*p* < 0.001)	−0.36 (−0.58 to −0.13, *p* = 0.002)		
Aldosterone				
Basal aldosterone	−0.09 (*p* = 0.42)	−0.17 (−0.41 to 0.07, *P* = 0.16)	0.01 (*p* = 0.93)	−0.07 (−0.31 to 0.17, *p* = 0.57)
Post/Basal aldosterone	0.02 (*p* = 0.89)	−0.04 (−0.28 to 0.20, *p* = 0.76)	−0.07 (*p* = 0.58)	0.12 (−0.36 to 0.13, *p* = 0.35)
Catecholamines				
Basal adrenaline	−0.09 (*p* = 0.43)	−0.17 (−0.41 to 0.07, *p* = 0.17)	0.01 (*p* = 0.93)	0.03 (−0.22 to 0.28, *p* = 0.80)
Basal noradrenaline	0.02 (*p* = 0.86)	−0.06 (−0.30 to 0.18, *p* = 0.62)	0.03 (*p* = 0.78)	0.07 (−0.17 to 0.32, *p* = 0.56)
Basal metanephrine	−0.08 (*p* = 0.54)	−0.15 (−0.39 to 0.09, *p* = 0.21)	0.02 (*p* = 0.90)	−0.03 (−0.28 to 0.21, *p* = 0.80)
Basal normetanephrine	0.15 (*p* = 0.22)	0.08 (−0.16 to 0.32, *p* = 0.50)	0.17 (*p* = 0.17)	0.16 (−0.08 to 0.40, *p* = 0.19)
Post/Basal adrenaline ratio	−0.16 (*p* = 0.20)	0 (−0.24 to 0.25, *p* = 0.97)	−0.17 (*p* = 0.17)	−0.03 (−0.28 to 0.21 *p* = 0.79)
Post/Basal noradrenaline ratio	−0.03 (*p* = 0.83)	−0.10 (−0.34 to 0.14, *p* = 0.43)	−0.15 (*p* = 0.21)	−0.20 (−0.44 to 0.04, *p* = 0.10)
Post/Basal metanephrine ratio	0.01 (*p* = 0.98)	−0.07 (−0.31 to 0.18, *p* = 0.55)	−0.10 (*p* = 0.43)	−0.10 (−0.34 to 0.14, *p* = 0.42)
Post/Basal normetanephrine ratio	−0.21 (*p* = 0.07)	−0.22 (−0.45 to 0.02, *p* = 0.07)	−0.27 (*p* = 0.03)	−0.27 (−0.50 to −0.03, *p* = 0.02)

### 3.5 Integrated multivariable model

A multivariable regression model was constructed to identify independent predictors of better performance, defined as a shorter marathon completion time. As summarized in Table 5, independent predictors of shorter marathon completion time were the change in normetanephrine levels, the change in the cortisol-to-cortisone ratio, lower minimum nocturnal heart rate and higher 2LV values. Among basal characteristics, only BMI but not training load or age remained independent predictors of shorter marathon finish time.

**Table 5:** Multivariable regression analysis for independent predictors of lower marathon completion time.

Variable	Standardized *β*	95% CI	*p*
Age	0.15	−0.05 to 0.35	0.13
BMI	0.20	0.02–0.37	0.03
Hours of training/week	−0.15	−0.32 to 0.02	0.10
Post/basal Normetanephrine ratio (hourly output)	−0.18	−0.35 to −0.05	0.02
Post/basal Cortisol/cortisone ratio	−0.22	−0.39 to −0.07	0.01
Minimum nocturnal HR	0.42	0.23 to 0.60	<0.001
2LV	−0.32	−0.52 to −0.13	0.001
Model *R*^2^	0.48		

BMI, body mass index; HR, heart rate; 2LV, 2 like variations.

### 3.6 Other findings

On the morning of the competition, the majority of athletes (77/104, 74%) reported caffeine intake, with a mean dose of 70 mg per person, consumed as coffee or supplements. Among these, 43 athletes used caffeine-containing supplements, with a mean dose of 100 mg and a maximum reported dose of 390 mg. Five athletes reported taking taurine-containing supplements, with a mean dose of 395 mg and a maximum dose of 1,500 mg. Twenty-seven athletes reported no intake of stimulants prior to the race. During the race, among the 57 athletes with ECG recordings of sufficient signal quality for more than 75% of the race duration, 24 exhibited at least one premature ventricular complex (PVC), whereas only 10 showed ≥3 PVCs. No complex ventricular arrhythmias were recorded during the race, and all PVCs were asymptomatic. No statistically significant association was observed between stimulants intake and the occurrence of exercise-induced PVCs (*p* = 0.9). However, the 20% of athletes with PVCs who consumed caffeine during the race had taken a higher median dose (150 mg vs. 114 mg) compared with the rest of the cohort. Finally, one athlete exhibited two episodes of regular paroxysmal supraventricular tachycardia at post-race analysis, lasting 9 and 4 min, respectively, with a maximum heart rate of 241 bpm. The arrhythmia was asymptomatic.

## 4 Discussion

The aim of this study was to investigate whether autonomic modulation and adrenal hormonal response were related to marathon finishing time, independently from personal characteristics such as age, BMI, and training history, in a cohort of Caucasian male master athletes.

The study was focused on a population of male master athletes in order to select a physiologically homogeneous cohort, since both age and sex are well-known determinants of hormonal secretion and autonomic modulation, potentially introducing significant variability in mixed populations. Male master athletes represent an interesting and relatively underexplored population. Most previous studies in exercise physiology have focused predominantly on young elite athletes, whereas non-elite master athletes have received comparatively little attention. At the same time, this group likely reflects the typical profile of participants in contemporary endurance events, where a large proportion of runners are middle-aged recreational athletes rather than elite competitors (24).

Analyses showed that several HRV and hormone variables were associated with better performance at univariate analysis, although *R*-values indicated weak-to-moderate relationships. Lower minimum nocturnal heart rate and vagal predominance, expressed as higher 2LV values, the ability to increase norepinephrine secretion, expressed as a higher post-/pre-race normetanephrine hourly production, and to transform cortisone into cortisol during exercise, expressed as post/pre-race shift in the cortisol/cortisone ratio, remained independent predictors at multivariable analysis. Only BMI, but not age and training history, remained significant in a model integrating HRV and hormones. This finding is consistent with the concept that autonomic nervous system and adrenal gland readiness for exercise, reflecting intrinsic physiological characteristics and training quality, may exert a greater influence on endurance performance than training quantity alone. However, the model showed an *R*^2^ value of 0.48, indicating a moderate goodness-of-fit and suggesting that other factors not included in the model contributed to athletic performance.

### 4.1 Autonomic nervous system modulation

During physical exercise, sympathetic activity increases at the expense of parasympathetic modulation through the so-called “central command,” leading to elevations in heart rate, blood pressure, and regional vascular resistance in order to meet the metabolic demands of working muscles (1, 3, 6, 25). This response is supported by the release of epinephrine from the adrenal medulla and norepinephrine from sympathetic nerve terminals at the cardiac level (25). Conversely, recovery from exercise is characterised by a progressive reactivation of parasympathetic tone (1, 3, 6, 25).

Enhanced parasympathetic modulation has historically been associated with training adaptations, including lower resting heart rate, increased stroke volume, and improved myocardial metabolic efficiency. Resting HRV is widely recognized as a marker of autonomic regulation, with higher variability generally reflecting greater vagal modulation, and has been associated with better training status in a number of previous studies. However, such previous research was mostly derived from controlled experimental protocols and relatively small cohorts (2, 26–28). In this study, HRV was evaluated and data obtained under real-life conditions in a cohort of middle-aged men running a marathon. It is important to underline that a greater parasympathetic modulation during sleep could also reflect a better sleep quality, which is a key determinant of recovery processes, hormonal balance and overall physiological readiness. In this context, enhanced nocturnal vagal modulation could represent not only a marker of training adaptation, but also an indirect indicator of more effective restorative sleep, ultimately contributing to improved performance outcomes (29–31).

In this real-life study focused on middle-aged male athletes, the athletes’ nocturnal HRV as a baseline parameter, therefore at rest, was considered, and it was found that a number of HRV parameters were associated with faster marathon finishing times. Among symbolic HRV parameters, higher 2LV and lower 0 V during sleep before the race were associated with faster marathon completion. Because these indices predominantly reflect parasympathetic and sympathetic modulation respectively, these findings support the concept that predominance of vagal modulation at rest is associated with better endurance performance. This predominance of vagal activity during the night may then further indicate a more efficient recovery state and superior sleep quality which in turn could enhance metabolic and cognitive preparedness for prolonged physical effort (29, 30). Higher SDNN and RMSSD, both time-domain HRV indices, were also associated with better performance. Because HRV-derived indices reflect partially overlapping aspects of autonomic modulation and are known to be strongly intercorrelated, they were not simultaneously included in the multivariable model in order to avoid multicollinearity and model overfitting. The parameter 2LV emerged as the best univariate predictor of finishing times and was therefore selected to be included in the multivariable model, where it remained independently associated with finishing time.

Another variable that emerged as an independent predictor of finishing times is the minimal nocturnal heart rate. Sinus bradycardia is a typical feature of the so-called athlete's heart and has traditionally been attributed to high vagal modulation. However, more recent evidence suggests that exercise training may also induce intrinsic modifications of sinus node cells, contributing to resting bradycardia not solely explained by vagal modulation (32, 33). Both mechanisms may reflect better physiological adaptation to training, leading to lower resting heart rate and parasympathetic dominance. Although sleep quality was not directly assessed, it is interesting to notice that a lower nocturnal heart rate, in combination with higher vagal modulation, may also be interpreted as a marker of deeper and more restorative sleep, further supporting the hypothesis that sleep-related autonomic patterns may contribute to endurance performance (29, 30).

### 4.2 Adrenal hormone response

Among the analysed hormones, only higher urinary post-race normetanephrine levels compared to baseline and a higher shift in the cortisol/cortisone ratio were independently associated with performance.

Normetanephrine is a more stable metabolite of norepinephrine and its level reflects more accurately norepinephrine production over longer time periods, such as the duration of a marathon. While epinephrine is produced by the adrenal gland, norepinephrine is the neurotransmitter released by sympathetic terminations and therefore higher normetanephrine levels could be interpreted as indicative of a more responsive sympathetic system capable of efficiently supporting cardiovascular and metabolic demands during exercise. A greater post- to pre-race increase in urinary normetanephrine was independently associated with shorter marathon finishing time: this finding is consistent with previous studies reporting an association between exercise-induced increases in plasma normetanephrine and running performance (34–36). This finding is also coherent with the predictive value of HRV indexes of parasympathetic tone at rest, suggesting the importance of ample autonomic system modulation (high vagal modulation at rest, high sympathetic tone during exercise) in supporting the athletic performance.

Physical exertion induces an increase in cortisol secretion, reflecting acute stress responses as well as potentially adaptive changes in tissue glucocorticoid sensitivity (9, 37, 38). Alongside cortisol, cortisone represents a key component of glucocorticoid metabolism. Cortisone acts as an inactive reservoir of cortisol and can be rapidly converted into its active form at the tissue level through 11β-hydroxysteroid dehydrogenase type 1, allowing a prompt increase in local glucocorticoid availability that is faster than *de novo* cortisol synthesis (7, 37). Accordingly, urinary cortisol and cortisone excretion, as well as the cortisol-to-cortisone ratio have been proposed as markers of training load, fatigue, and recovery status. From a physiological perspective, the cortisol-to-cortisone ratio may reflect the balance between glucocorticoid demand and the mobilization of endogenous cortisone reserves, thereby providing an integrated measure of stress burden and glucocorticoid utilization.

This study identified an association between a higher shift in the cortisol/cortisone ratio and performance, reflecting the higher ability of faster runners to convert cortisone into cortisol through 11β-hydroxysteroid dehydrogenase type 1 during the marathon. This finding indicates a greater metabolic readiness and a more efficient endocrine response to exercise stress.

### 4.3 Other findings and general considerations

Stimulant use was common, and a higher occurrence of PVCs during exercise was observed among athletes who reported higher caffeine intake during the race, although overall ventricular arrhythmia prevalence remained low. This finding contrasts with recent reports suggesting a higher prevalence of exercise-induced ventricular arrhythmias in master athletes (39, 40). However, it should be emphasized that preparticipation medical evaluation is mandatory for marathon runners in Italy, and athletes with significant exercise-induced arrhythmias related to an underlying cardiac disease may therefore have been excluded *a priori* from the competition (41).

### 4.4 Strengths, limitations and further directions

Several limitations should be acknowledged. Standardization of urine sampling was challenging due to the field-based design of the study, and urinary hormone measurements reflect integrated secretion over time rather than precise temporal changes during exercise. HRV measurements were obtained during a single pre-race night and may therefore have been influenced by situational factors such as pre-race stress or sleep quality. However, the sleep period was selected for baseline HRV analysis because the hours before the race were marked by significant fluctuations in activity level. ECG recording was performed using a single-lead device, which increased the risk of artefacts; consequently, many recordings had to be excluded because of insufficient quality. The study population consisted exclusively of Caucasian male master athletes, which may limit the generalizability of these findings to female athletes, younger competitors, elite runners, or other ethnic groups. However, despite these limitations, this study is the first to comprehensively investigate, in a real-world setting, autonomic and endocrine responses to strenuous exercise in a relatively large cohort of trained athletes who had undergone pre-participation screening, integrating these parameters into a multivariable model.

These findings may contribute to a better understanding of the complex interplay between hormonal and autonomic parameters and athletic performance. Nevertheless, further studies in larger cohorts, including women and athletes from diverse ethnic backgrounds, are warranted to validate these findings. In addition, the identification of additional variables contributing to performance, together with an assessment of their relative contribution, may help refine the predictive model and provide deeper insight into the physiological determinants of performance.

## 5 Conclusions

In a cohort of male master athletes participating in a marathon, autonomic cardiovascular control during the sleep period of the night before the race (considered as a baseline) and endocrine responsiveness during exercise emerged as potential predictors of performance. Higher vagal modulation during the pre-race night, lower nocturnal heart rate, and a more pronounced activation of sympathetic activity and cortisone-to-cortisol conversion during exercise were independently associated with faster marathon finishing times.

These results support the potential role of training strategies and lifestyle habits aimed at optimizing autonomic regulation and recovery, including appropriate training quality, adequate sleep, and healthy lifestyle behaviours. However, it is important to stress that because of the observational design of the study, causal relationships between autonomic modulation, endocrine responses, and marathon performance cannot be established. Moreover, the moderate goodness-of-fit of the multivariable model suggests that marathon performance is influenced by other factors, including pacing strategy, nutritional intake, and environmental conditions, which could not be fully controlled in this real-life setting.

## Data Availability

The raw data supporting the conclusions of this article will be made available by the authors, without undue reservation.
